# IL-2 Complex Therapy Mitigates Humoral Rejection of Fully Mismatched Skin Allografts by Inhibiting IgG Alloantibody Formation

**DOI:** 10.3390/cells14141086

**Published:** 2025-07-16

**Authors:** Konstantinos Mengrelis, Mario Wiletel, Romy Steiner, Anna M. Weijler, Laurenz Wolner, Valentina Stolz, Milos Nikolic, Daniel Simon, Florian Frommlet, Jonathan Sprent, Hannes Stockinger, Nina Pilat

**Affiliations:** 1 Department of Cardiac and Thoracic Aortic Surgery, Medical University of Vienna, 1090 Vienna, Austriaromy.steiner@meduniwien.ac.at (R.S.); 2 Department of General Surgery, Division of Transplantation, Medical University of Vienna, 1090 Vienna, Austriaanna.weijler@meduniwien.ac.at (A.M.W.);; 3 Center for Biomedical Research and Translational Surgery, Medical University of Vienna, 1090 Vienna, Austria; laurenz.wolner@meduniwien.ac.at; 4 Division of Immunobiology, Institute of Immunology, Center for Pathophysiology, Infectiology and Immunology, Medical University of Vienna, 1090 Vienna, Austria; 5 Center for Medical Statistics, Informatics and Intelligent Systems, Section for Medical Statistics, Medical University of Vienna, 1090 Vienna, Austria; 6 Garvan Institute of Medical Research, Immunology Division, Sydney, NSW 2010, Australia; j.sprent@garvan.org.au; 7 St Vincent’s Clinical School, University of New South Wales, Sydney, NSW 2010, Australia; 8 Center for Pathophysiology, Infectiology and Immunology, Institute for Hygiene and Applied Immunology, Medical University of Vienna, Vienna 1090, Austria; hannes.stockinger@meduniwien.ac.at

**Keywords:** transplantation, regulatory T cells, germinal center, donor-specific antibodies, humoral tolerance

## Abstract

Antibody-mediated rejection (ABMR) caused by donor-specific Abs (DSAs) is still the leading cause of late graft loss following clinical organ transplantation, and effective strategies to combat ABMR are still elusive. We previously showed that rIL-2 complexed with anti-IL-2 mAb clone JES6-1A12 (IL-2 cplx) leads to the selective expansion of regulatory T cells (Tregs) and the prolonged survival of MHC-mismatched skin allografts. Although the grafts were eventually rejected, mice failed to develop DSAs. Here, we investigated the impact of IL-2 cplx on the humoral response and germinal center (GC) reaction during allograft rejection. IL-2 cplx treatment prevents Bcl-6 upregulation, leading to suppressed development of GC T and B cells. The IL-2 cplx-induced impairment of GC development limits IgG allo-Ab production but allows for IgM synthesis. By employing a hapten–carrier system to investigate affinity maturation, we found that IL-2 cplx induces a distinct shift in specific Ab production favoring low-affinity IgM while simultaneously decreasing IgG responses. These findings illuminate the potential of IL-2 cplx therapy for inducing humoral tolerance, potentially paving the way for refining strategies aimed at preventing and treating ABMR.

## 1. Introduction

Despite progress in effective immunosuppressive therapy, Ab-mediated rejection (ABMR) is the most common cause of immune-mediated allograft failure after kidney transplantation and impedes long-term survival [1]. Hence, there is a pressing need to devise new protocols for impairing allo-Ab production following transplantation. Additionally, as well as eliciting chronic allograft vasculopathy, donor-specific HLA Abs can develop de novo soon after transplantation and trigger acute ABMR in some recipients. Importantly, de novo ABMR is not responsive to traditional anti-lymphocytic treatments [2]. In addition, preformed Abs against diverse antigens restrict access to organs and induce prolonged waiting times. Effective clinical treatment strategies for the tailored prevention of donor-specific Ab (DSA) formation are still not available. In preclinical animal models adoptive cell therapy with therapeutic T regulatory cells (Tregs) has been shown to prevent allograft vasculopathy [3], and Treg-enhancing therapies may prevent germinal center (GC) allo-Ab responses [4].

At low doses, the pleiotropic cytokine IL-2 induces the selective expansion of CD4+ CD25+ FoxP3+ Tregs in vivo due to their high expression of the high-affinity IL-2 receptor, composed of the IL-2Rα (CD25) chain, the IL-2Rb (CD122) chain and the IL-2Rg (CD132) chain [5]. As a result, the induction of immunological tolerance by low-dose rIL-2 has been explored for the treatment of graft-versus-host disease (GVHD) [6], autoimmune disorders [7] and rejection in solid organ transplantation [8]. Two major clinical limitations of soluble rIL-2 administration are (1) diverse off-target effects, orchestrated by immune populations expressing distinct IL-2R subunits, and (2) the short bioavailability and half-life of rIL-2 in vivo. These problems can be bypassed by complexing rIL-2 with certain anti-IL-2 mAbs. IL-2/anti-IL-2 complexes exhibit an extended half-life and enhanced bioavailability of rIL-2. Strikingly, IL-2 complexes formed with the specific mAb JES6-1A12 (referred to as IL-2 cplx hereafter) block IL-2 interaction with the intermediate-affinity IL-2 receptor (composed of CD122 and CD132) found on, e.g., memory T cells and NK cells, but do not impede IL-2 interaction with CD25 [9]. Consequently, IL-2 cplx promotes the expansion of, preferentially, Tregs expressing the high-affinity IL-2 receptor (composed of CD25, CD122 and CD132) and, as result, upon injection in mice, the onset of immunosuppression [9]. In contrast, another mAb, namely, S4B6, forms complexes with IL-2 that mainly act on memory phenotype (MP) CD8 T cells and NK cells, akin to high-dose IL-2 therapy [9].

The selective expansion of Tregs via IL-2 cplx administration has previously been explored for the induction of peripheral tolerance in murine models. Combined with rapamycin, the complexes prevented the induction of experimental autoimmune encephalomyelitis (EAE) [10] and arthritis [11]. In a transplantation setting, short-term IL-2 cplx treatment led to the indefinite survival of >80% of islet allografts [10]. Recently, IL-2 cplx treatment was shown to synergize with rapamycin and short-term IL-6 blockade, leading to significantly prolonged survival of fully mismatched (BALB/c → B6) skin allografts [12] in the absence of ongoing treatment. Interestingly, although the grafts were eventually rejected, the sera of IL-2 cplx-treated mice were almost devoid of anti-donor IgG Abs and a second-set skin graft from the same donor was rejected with the same kinetics as in naïve mice, indicating that IL-2 cplx treatment impaired both humoral and cellular immunity [12]. We hypothesized that the inhibition of DSAs was primarily mediated by IL-2 cplx treatment, as it was also seen in mice treated with IL-2 cplx alone but not in mice treated with rapamycin and anti-IL-6 (in which case the mice developed normal levels of DSAs [12].

Since IL-2 therapy works well for transplantation tolerance in animal models, it is important to have a clear understanding of the mechanisms of tolerance involved. For treatment with IL-2 cplx [9], low-dose soluble IL-2 [13,14], or IL-2 mutein [15], tolerance is known to reflect the stimulation and expansion of CD25+ Tregs [9,16]. However, precisely how the stimulation of these cells impairs allo-Ab production—and Ab production in general—is basically unknown and a topic of intense investigation. Recently, it was shown that a humanized mutein IL-2 selectively promotes Treg expansion in preclinical transplantation models. The translational potential was underlined by in vivo Treg expansion in cynomolgus monkeys and humanized mouse models [15].

In this study, we investigated the effects of IL-2 cplx treatment on T- and B-cell subsets within secondary lymphoid organs of mice carrying an MHC-mismatched skin graft. Our results show that IL-2 cplx-induced skin-graft tolerance resulted in an impaired GC response, affecting the generation of T- and B-cell subsets. Specifically, IL-2 cplx exposure during the immune response suppressed the generation of both T follicular helper (Tfh) and T follicular regulatory (Tfr) cells within the GC, despite its stimulatory effects on Tregs. This treatment also significantly decreased allo-IgG production while not significantly affecting allo-IgM levels. Furthermore, in a hapten–carrier system, we observed that IL-2 cplx injection increased or maintained normal levels of somatic hypermutation (SHM) for IgG Abs but markedly reduced the binding strength of IgM Abs.

## 2. Materials and Methods

### 2.1. Mice and Study Approval

Age-matched female C57BL/6 (B6; recipient, H-2b) and BALB/c (donor, H-2d) mice were purchased from Charles River Laboratories (Sulzfeld, Germany). All mice were used at the age between 6 and 10 weeks with an average weight of 18–20 g. In these experiments, female mice were used. Mice were housed under SPF conditions in individually ventilated filter cages (up to 5 mice per cage; randomized at the start of the experiment) on sterile standard bedding; sterile water and a standard pellet diet were given ad libitum. Housing rooms were under a 12 h light cycle. All experiments were discussed and approved by the Ethics and Animal Welfare Committee of Medical University of Vienna and were performed in strict accordance with national and international guidelines of laboratory animal care. All animals received humane care in compliance with FELASA and ARRIVE and are covered by ethics vote of the Austrian Federal Ministry of Science, Research and Economy. Experiments were approved by the Austrian Federal Ministry of Science, Research and Economy (BMBWF-66.009_0237-V_3b_2018, GZ 2023-0.315.081). All surgeries were performed under general anesthesia employing a mixture of ketamine (100 mg/kg) and xylazine (5 mg/kg) i.p., and postoperative analgesia consisted of buprenorphin (Buprenovet, on d 0; 0.01–0.05 mg/kg/d, i.p.), followed by piritramid (Dipidolor, 15 mg in 250 mL of 0.4% glucose water) in drinking water ad libitum for 1 week. The mice were submitted to euthanasia by cervical dislocation as requested by the ethics votum. The concept of 3Rs (replacement, refinement and reduction) was implemented in the study design of the approved ethical protocol. All efforts were made to minimize distress and group size. The number of mice in each specific experimental group is provided in the figure legend. Experimental groups were age-matched, and information about littermates was not available from the supplier.

### 2.2. Sex as a Biological Variable

Our study exclusively examined female animals, allowing for group housing (instead of individual housing, which is often required for male C57BL/6 mice) to reduce the stress for the animals in the interest of implementing the 3R principles. Similar findings have already been reported for both sexes in similar studies in mice [15]; therefore, results regarding basic mechanistic questions in this model are expected to be relevant for male and female mice. We are aware that clinical translation will need preclinical verification in both male and female mice especially with relation to dosing regimens; additionally, possible differences need to be assessed for other factors associated with age and strain/ethnicity.

### 2.3. IL-2 Cplx Preparation

**Figure 1:**
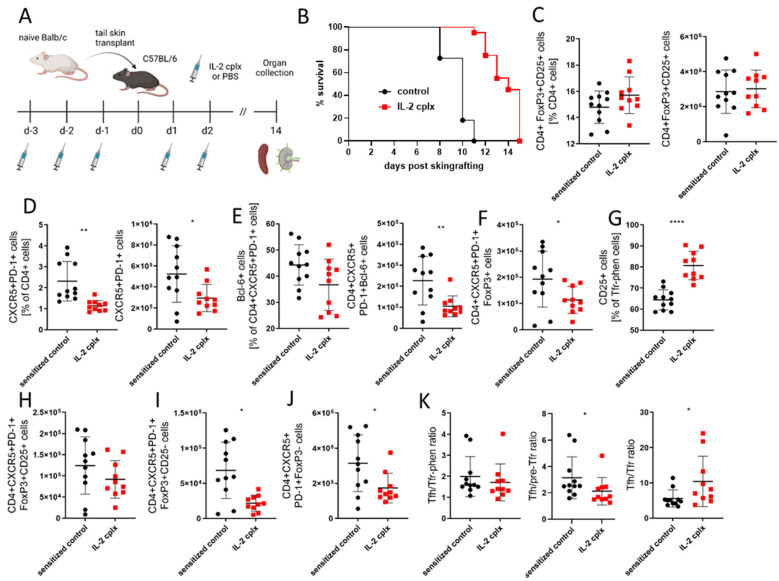
JES6-1A12 IL-2 cplx impairs germinal center T-cell differentiation in the spleen of grafted mice. Fully mismatched BALB/c skin-grafted C57BL/6 mice were treated with either PBS (sensitized control, *n* = 11) or JES6-1A12 IL-2 cplx (IL-2 cplx, *n* = 10). Changes in leukocyte subsets were analyzed on day 14. (**A**) Schematic illustration of experimental setup (created in BioRender; Pilat-michalek, N. (2025) https://BioRender.com/mkqvnef). (**B**) Survival curves of cumulative data of two independent experiments are shown. (**C**) shows the proportion, gated on CD4+ T cells, and absolute cell count, respectively, of CD25+FoxP3+ cells recovered from the spleens of the mice. (**D**) depicts the proportion, gated on CD4+ T cells, and absolute count, respectively, of CXCR5+PD1+ splenocytes. (**E**) shows the proportion of CD4+CXCR5+PD1+ splenocytes that stain positive for intracellular Bcl-6, and the absolute count of CD4+CXCR5+PD1+Bcl-6+ cells (GC T cells). (**F**) The absolute count of CD4+CXCR5+PD1+FoxP3+ cells (Tfr-phen) is shown. (**G**) shows the proportion of CD25-expressing Tfr-phen in the spleens. The absolute counts of (**H**) CD4+CXCR5+PD1+FoxP3+CD25+ cells (pre-Tfr) and (**I**) CD4+CXCR5+PD1+FoxP3+CD25-cells (Tfr) are shown. The absolute count of CD4+CXCR5+PD1+FoxP3-cells (Tfh) is given in (**J**). (**K**) depicts the ratios of splenic Tfh to either Tfr-phen, pre-Tfr or Tfr cells. In all scatter plots in this figure, each point represents an individual mouse, and bars show mean ± SD (data are pooled from 2 independent experiments), * *p* < 0.05, ** *p* < 0.01, and **** *p* < 0.0001.

IL-2/anti–IL-2 mAb complexes were prepared as previously described [9,10]. Briefly, mouse rIL-2 (PeproTech, Hamburg, Germany) was mixed with purified anti-mouse IL-2 mAb (JES6-1A12, or S4B6-1 for indicated experiments; BioXcell, West Lebanon, NH, USA) in a molar ratio of 1:5 and incubated at 37 °C for 30 min [9]. IL-2 cplx was administered i.p. (1 μg in a 200 µL final volume of PBS) on indicated days (Figure 1A, Supplementary Figure S1a).

### 2.4. Flow Cytometric Analysis and mAbs

Sample preparation and staining were conducted as described previously [12]. Spleens and lymph nodes were harvested at indicated timepoints, and single-cell suspensions were prepared. Red blood cell lysis in spleens was performed using Hybri-Max buffer (Sigma-Aldrich/Merck, Darmstadt, Germany) according to the manufacturer’s protocol. For the analysis of B- and T-cell subpopulations the following fluorescent anti-mouse mAbs were used: CD45R/B220 (RA3-6B2; RRID: AB_394335), IgM (R6-60.2; RRID: AB_2872206), IgD (11-26c.2a; RRID: AB_10612002), IgG1 (A85-1; RRID: AB_1645625), IgG2a (R19-15; RRID: AB_2742304), IgG3 (R40-82; RRID: AB_394840), CD23 (B3B4; RRID: AB_2737820), CD19 (1D3; RRID: AB_1645234), CD1d (1B1; RRID: AB_2743723), T- and B-Cell Activation Antigen (GL7; RRID: AB_10716056), CD138 (281-2; RRID: AB_2871390) and Fas/CD95 (Jo2; RRID: AB_2917330), TACI (8F10-3,; RRID: AB_2741091) and Fixable Viability Stain 575V—all BD Pharmingen (BD); further, we used CD3 (17A2, BioLegend; RRID: AB_2561455), CD4 (GK15, eBioscience/ThermoFisher Scientific, Bremen, Germany; RRID: AB_467063), CXCR5 (L138D7, BioLegend; RRID: AB_2566798), CD44 (IM7, Thermofisher Scientific, Bremen, Germany; RRID: AB_465045), CD25 (PC61.5, eBioscience; RRID: AB_1272179), MHC-class II (M5/114.15.2, eBioscience; RRID: AB_10870792), PD-1 (RMP1-30, eBioscience; RRID: AB_466290) and PD-L1 (MIH5, eBioscience; RRID: AB_466089). Cell suspensions were incubated for 10 min with purified anti-mouse CD16/32 (2.4G2, BD; RRID: AB_394656) to block unspecific Fc binding and then stained for surface markers for 30 min. Intracellular FoxP3 (FJK-16s, eBioscience; RRID: AB_469457), Blimp-1 (5E7, BD; RRID: AB_2738719), Bcl-6 (K112-91, BD Biosciences, Heidelberg, Germany; RRID: AB_11152084) and IRF4 (3E4, Biolegend; RRID: AB_2814496) staining assays were performed with either the BD Transcription factor buffer set or the eBioscience Fixation/Permeabilization kit according to the manufacturers’ instructions. Cells were acquired with a BD Fortessa LSRII or a BD Canto II and analyzed with FlowJo V8. The flow cytometric markers for the analysis of B- and T-cell subpopulations are listed in Table 1, and the gating strategies are provided in Supplementary Figure S3A–D.

### 2.5. MHC-Specific ELISA

Serum from skin-grafted mice was harvested 14 d after skin engraftment and analyzed for donor-specific IgG isotypes and IgM against rMHCII I-Ad monomers. Maximal-absorbance 96-well plates were coated overnight with 5 μg/mL monomers (100 μL/well), kindly provided by the NIH Tetramer Core Facility (Atlanta, GA, USA). Serum (1 μL) was diluted to 1:100 in PBS-T (PBS 0.05% Tween 20)/BSA, and bound Abs were detected by indirect staining using monoclonal rat anti-IgM (R6-60.2; RRID:AB_394842), anti-IgG1 (A85-1; RRID:AB_394860) or anti-IgG2a (R19-15; RRID:AB_394825) Ab (all BD) diluted to 1:1000 and HRP-coupled goat anti-rat antiserum (eBioscience/ThermoFisher Scientific, Bremen, Germany) diluted to 1:2000 in PBS-T/BSA. 2,2′-azino-bis(3-ethylbenzothiazoline-6-sulfonic acid (ABTS) was used as the substrate for HRP. Absorbance measurements were taken with a microplate reader (Infinite F50, TECAN, Grödig, Austria).

### 2.6. NP-KLH Immunization

Mice were immunized by left-footpad injection with 10 µg of antigen 4-hydroxy-3-nitrophenylacetyl-keyhole limpet hemocyanin (NP-KLH, Biosearch Technologies, Petaluma, CA, USA) in 100 µL of Imject alum adjuvant (Pierce Biotechnology, Rockford, IL, USA). PE (Sigma-Aldrich/Merck, Darmstadt, Germany) was coupled to NP by reacting with 40 μg of NP-O-suc (Biosearch Technologies Inc.) in dimethyl formamide (Sigma-Aldrich/Merck, Darmstadt, Germany) in 0.2 M carbonate/bicarbonate buffered to pH 9.0.

### 2.7. Affinity Binding Assay

Mouse serum was analyzed on days 7 and 14 post-NP-KLH immunization for the levels of NP-specific IgG1 and IgM serum Abs using two different coupling ratios of NP-BSA as described [28]. Briefly, NP2-BSA or NP23-BSA (10 μg/mL; Biosearch Technologies, Petaluma, CA, USA) were coated onto 96-well ELISA plates, and serum was added in serial dilutions for 2 h at room temperature (RT). Plates were washed to remove unbound serum, and goat anti-mouse HRP (Southern Biotechnology, Birmingham, AL, USA)-conjugated isotype-specific secondary Abs against mouse IgM or IgG1 were added as previously described [29]. The ratio between NP2 and NP23 binding Abs was calculated as an estimate of affinity maturation [30,31].

### 2.8. Skin Grafting

Full-thickness tail skin from BALB/c mice was grafted on the side of the chest of C57BL/6 recipients, secured with stitches and band aids for 6 d and visually inspected thereafter at short intervals. Grafts were considered to be rejected when less than 10% remained viable.

### 2.9. Anti-Donor Flow Cytometric Crossmatch

Heat-inactivated recipient serum harvested 14 d after skin-graft transplantation was incubated with recipient-type and donor-type thymocytes (mostly MHCI-positive) and splenocytes (MHCI- and MHCII-positive), respectively. The binding of serum IgG Abs to thymocytes and splenocytes was analyzed by flow cytometry using monoclonal rat anti-mouse IgG1 (RMG1-1; Biolegend, RRID: AB_10696420), IgG2ab (R2-40; BD Biosciences, RRID: AB_2743817) and IgG3 (R40-82; BD Biosciences, RRID: AB_394840); the gating strategies are provided in Supplementary Figure S3E.

### 2.10. Statistical Analysis

Data analysis was performed with GraphPad Prism 8.0 (GraphPad Software) using an unpaired 2-tailed t-test to compare differences between two groups. For comparison of 3 or more unpaired groups, 1-way ANOVA was used. Skin-allograft survival was calculated according to the Kaplan–Meier product limit method and compared between groups using the log-rank test. A *p*-value < 0.05 was considered to denote statistical significance (* *p* < 0.05, ** *p* < 0.01, *** *p* < 0.001 and **** *p* < 0.0001).

## 3. Results

### 3.1. IL-2 Cplx Treatment of MHC-Mismatched Skin-Grafted Mice Leads to Impaired GC T-Cell Formation

In order to determine the physiological consequences of IL-2 cplx treatment in the presence of alloantigens and sustained antigenic recognition, we examined T- and B-cell populations in fully mismatched skin-grafted mice (BALB/c-to-B6 strain combination). Mice were i.p. injected with IL-2 cplx on days −3/−2/−1/+1/+2, or with PBS as a control (Figure 1A); skin grafts were applied on day 0, and the mice were submitted to euthanasia on day 14. As shown previously [12], IL-2 cplx treatment alone was not sufficient for the induction of (operational) tolerance in a setting of fully mismatched skin allografts (in Figure 1B, MST days 10 vs. 13). However, it was already shown that a short course of IL-2 cplx (but neither rapamycin or anti-IL-6 alone or in combination) impairs the humoral response and the development of donor-specific IgG [12].

In accord with previous studies [5,12], we found that splenic Tregs increased significantly in IL-2 cplx-treated compared with untreated mice (Supplementary Figure S1A), both in percentages and numbers (Supplementary Figure S1B; 2 days after the last injection). In the skin transplant model, Treg percentages and numbers had returned to near-normal levels by the time of euthanasia (Figure 1C; 12 days after the last injection). However, unlike in ungrafted mice (Supplementary Figure S1C), both the proportion and absolute counts of splenic CD4 + CXCR5 + PD1 + GC T cells decreased 2-fold upon JES6-1A12 IL-2 cplx treatment in grafted mice (Figure 1D); this effect was specific for JES6-1A12 IL-2 cplx and was not seen in S4B6 IL-2 cplx. Within this cell compartment, Bcl-6 expression was lower in JES6-1A12 IL-2 cplx-treated mice, and although the change did not reach significance, the total numbers of CD4 + CXCR5 + PD1 + Bcl-6 + cells decreased 2.5-fold (Figure 1E; again, this is different in naïve mice, as shown in Supplementary Figure S1D). Typing these GC T cells for Foxp3 expression showed that IL-2 cplx injection caused a 2-fold decrease in the total numbers of Tfr-phen cells (Figure 1F; Supplementary Figure S1E shows a significant increase in FoxP3 expression in ungrafted mice). CD25 expression on Tfr-phen cells increased significantly after IL-2 cplx injection (Figure 1G). As a result, the numbers of pre-Tfr cells (CD25+) remained stable between the two groups, whereas the numbers of Tfr cells (CD25-) dropped significantly in the IL-2-cplx-treated group (Figure 1H,I). The numbers of Tfh cells also dropped significantly (Figure 1J). The Tfh/Tfr-phen ratio remained stable relative to untreated mice; however, the Tfh/pre-Tfr ratio decreased, whereas the Tfh/Tfr ratio increased significantly, relative to untreated mice (Figure 1K).

The main conclusion from these findings in the spleen is that unlike in naïve mice (Supplementary Figure S1A–J), JES6-1A12 IL-2 cplx injection in grafted mice caused a substantial reduction in the numbers of both Tfh and Tfr cells but not pre-Tfr cells (similar trends have been found in cells recovered from lymph nodes). Consequently, the reduction in Tfr cells was accompanied by an apparent delay in the transition of pre-Tfr cells into Tfr cells.

### 3.2. IL-2 Cplx Treatment of MHC-Mismatched Skin-Grafted Mice Leads to Impaired GC B-Cell Formation

**Figure 2:**
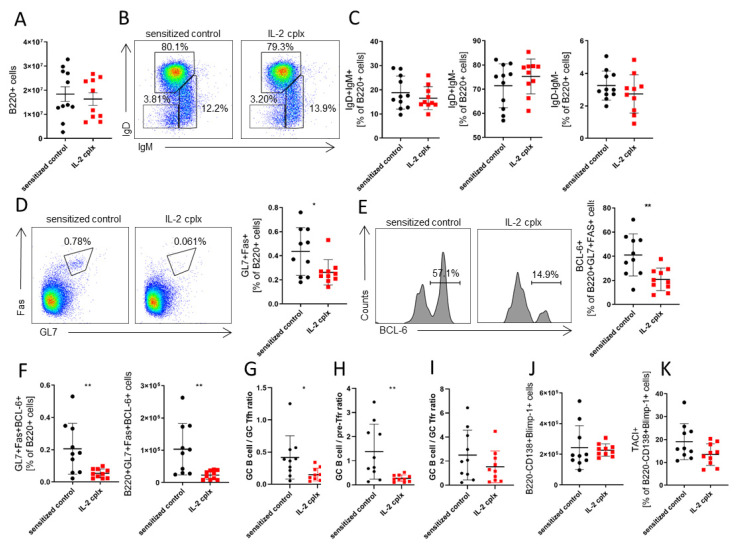
JES6-1A12 IL-2 cplx impairs germinal center formation in the spleen of skin grafted mice. Fully mismatched BALB/c skin grafted C57BL/6 mice were treated with either PBS (sensitized control, *n* = 11) or JES6-1A12 IL-2 cplx (IL-2 cplx, *n* = 10). Changes on leukocyte subsets were analyzed on day 14. (**A**) shows the absolute cell count of B220+ cells, gated on splenic lymphocytes. (**B**) Representative dot plots show anti-IgM against anti-IgD staining, gated on B220+ splenocytes. (**C**) Scatter plots show the proportion of IgD+IgM+, IgD+IgM-, IgD-IgM- B cells, respectively. (**D**) Representative FACS plots depict anti-Fas and anti-GL7 staining of B220+ splenocytes and the scatter plots show the proportion of B220+ cells that are Fas+GL7+. (**E**) Representative histograms (left) and scatter plot (right) depict intracellular Bcl-6 expression, gated on B220+GL7+Fas+ splenocytes. (**F**) Scatter plot shows the proportion of B220+ cells that are GL7+Fas+Bcl-6+ (GC B cells; left) and the absolute count of B220+GL7+Fas+Bcl-6+ GC B cells (right). (**G**–**I**) show the ratio of GC B cells (B220+GL7+Fas+Bcl-6+) to GC Tfh cells (CD4+CXCR5+PD1+FoxP3-), pre-Tfr cells (CD4+CXCR5+PD-1+FoxP3+CD25+) and GC Tfr cells (CD4+CXCR5+PD-1+FoxP3+CD25- cells). (**J**) depicts the absolute cell count of B220-CD19-CD3-CD138+Blimp1+ plasma cells whereas (**K**) shows the proportion of splenic plasma cells that are TACI+. In all scatter plots in this figure, each point represents an individual mouse, and bars show mean ± SD (data are pooled from 2 independent experiments; one sample of the control group is not reported in panels D-K as it was lost during acquisition), * *p* < 0.05, ** *p* < 0.01.

IL-2 cplx injection of skin-grafted mice did not change the counts of total B220+ B cells in the spleen (Figure 2A), their expression of IgD or IgM (Figure 2B,C) or the proportion of marginal zone (MZ) B cells, which is similar to the data obtained in naïve mice (Supplementary Figure S2A–C). However, we detected a significant decrease in the proportion and absolute numbers of activated GL7 + Fas + B cells in IL-2 cplx-treated skin-grafted mice (Figure 2D; no difference in IL-2 cplx-treated naïve mice, as shown in Supplementary Figure S2D); this finding was not apparent in S4B6 IL-2 cplx-treated mice. Therefore, a specific effect on CD25-expressing cell populations is suggested. A direct effect of IL-2 cplx treatment on B cells despite their relative lack of CD25 expression cannot be ruled out, as CD25 can be upregulated on B cells during allogeneic activation [32]. However, for GL7 + Fas + B cells, there was a parallel decrease in intracellular IRF4 and Bcl-6 expression levels in IL-2 cplx-treated mice, implying less localization of these B cells in GC (Figure 2E). Importantly, as indicated by B220 + GL7 + Fas + Bcl-6 + expression, GC B cells were substantially diminished by IL-2 cplx administration in alloantigen challenged mice, both in their proportion and total numbers (Figure 2F; no such changes could be observed in naïve mice; Supplementary Figure S2E,F). In the transplantation setting, IL-2 cplx treatment significantly increased the relative abundance of follicular T cells in relation to GC B cells (Figure 2G–I). Here, after IL-2 cplx injection, the mean ratio of GC B cells/GC Tfh decreased significantly (Figure 2G). Additionally, there was a significant decline in the GC B cell/pre-Tfr ratio (Figure 2H) and a non-significant reduction in the GC B cell/Tfr ratio (Figure 2I). IL-2 cplx injection in skin-grafted mice failed to alter the total numbers of typical plasma cells (Figure 2J) in contrast to naïve mice (Supplementary Figure S2H), though the levels of TACI on these cells were mildly reduced (Figure 2K).

Thus, IL-2 cplx treatment of skin-grafted mice caused a marked decrease in the total number of GC B cells, suggesting a reduction in GC reaction to donor alloantigens.

### 3.3. Circulating Alloantigen-Specific IgG but Not IgM Isotypes Are Significantly Decreased in Serum of Skin-Grafted Mice Treated with IL-2 Cplx

**Figure 3:**
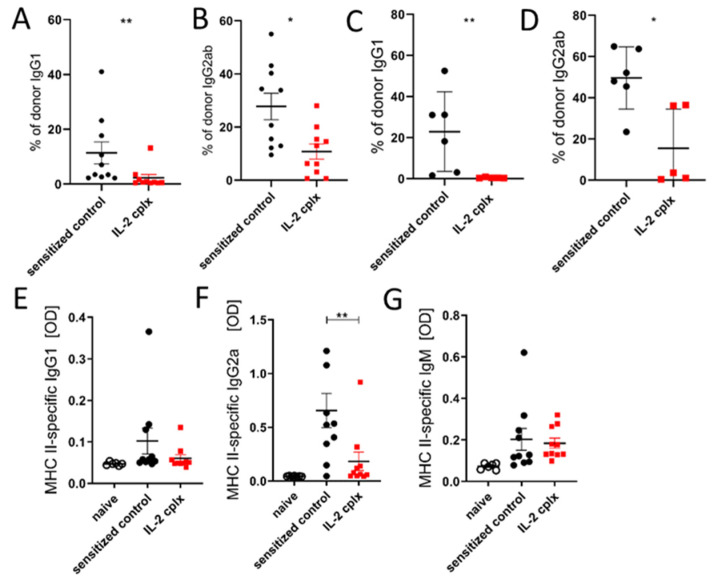
JES6-1A12 IL-2 cplx inhibits class switch in skin-grafted mice. Fully mismatched BALB/c skin-grafted C57BL/6 mice were i.p. injected with either PBS (sensitized control) or JES6-1A12 IL-2 cplx (IL-2 cplx). Fourteen days after skin transplantation, sera were harvested, and the development of donor-specific Ig was assessed by either flow cytometric crossmatch (**A**–**D**) or MHC II-specific ELISA (**E**–**G**). (**A**,**B**) Scatter plots showing the percentage of donor MHC class I-specific IgG1 and IgG2ab with donor-strain thymocytes and sera from untreated (*n* = 10) and JES6-1A12 IL-2 cplx-treated (*n* = 10) mice. (**C**,**D**) Scatter plots showing donor-specific IgG subtypes IgG1 and IgG2ab for MHC class I and class II after incubation of donor-strain splenocytes with sera from PBS-treated (untreated, *n* = 6) and JES6-1A12 IL-2 cplx-treated (*n* = 5) adult mice. MHC class II-specific IgG1 (**E**), IgG2a (**F**) and IgM (**G**) assessed by ELISA in naïve mice (*n* = 6) and in PBS-treated (sensitized, *n* = 10) and IL-2 cplx-treated (IL-2 cplx, *n* = 10) mice 14 days after skin engraftment. * *p* < 0.05, ** *p* < 0.01.

To examine whether the decreased formation of GC B cells induced by IL-2 cplx injection was antigen-specific, we measured the levels of alloantigen-specific Abs in the serum of the skin-grafted mice 14 days after skin-graft rejection. We used a flow cytometric assay that measured the binding specificity of recipient serum-derived Abs to donor-strain thymocytes (detecting mainly the humoral response against MHC class I [33]). IgG1 Abs were almost completely absent (Figure 3A), the mean IgG2ab levels were reduced 3-fold (Figure 3B), and IgG3 Abs were undetectable in both groups. Similar results were obtained when we used donor-strain splenocytes (expressing MHC classes I and II), showing the complete absence of donor IgG1 in IL-2 cplx-treated mice and a 3-fold reduction in IgG2ab (Figure 3C,D). An alloantigen-specific ELISA with rMHC-II I-Ad monomers revealed that the levels of MHC class II-specific IgG1 were slightly reduced and those of MHC class II-specific IgG2a significantly reduced in IL-2 cplx-treated skin-grafted mice compared with untreated skin-grafted controls (Figure 3E,F). By contrast, the relative level of donor-specific IgM Abs remained stable between IL-2 cplx-treated and untreated skin-grafted mice (Figure 3G). Interestingly, one mouse from the treated group showed high levels of IgG Abs, similar to the mean of the untreated group in both ELISA and crossmatch experiments. This mouse contained the lowest numbers of pre-Tfr—but not GC Tfr, GC Tfh or GC B—cells.

The above findings established that IL-2 cplx treatment of grafted mice caused a marked decrease in allo-Ab generation, but only for IgG and not for IgM. Since the SHM of B cells in GC applies to both IgM and IgG production [34], the question of whether IL-2 cplx treatment of grafted mice affected the affinity maturation of the responding alloreactive B cells arises. As affinity maturation is difficult to test for allo-Ab production, we addressed this issue indirectly with a hapten–carrier system as described below.

### 3.4. IL-2 Cplx Treatment Leads to Qualitative Changes in the Ab Response to a Hapten–Carrier Conjugate

**Figure 4:**
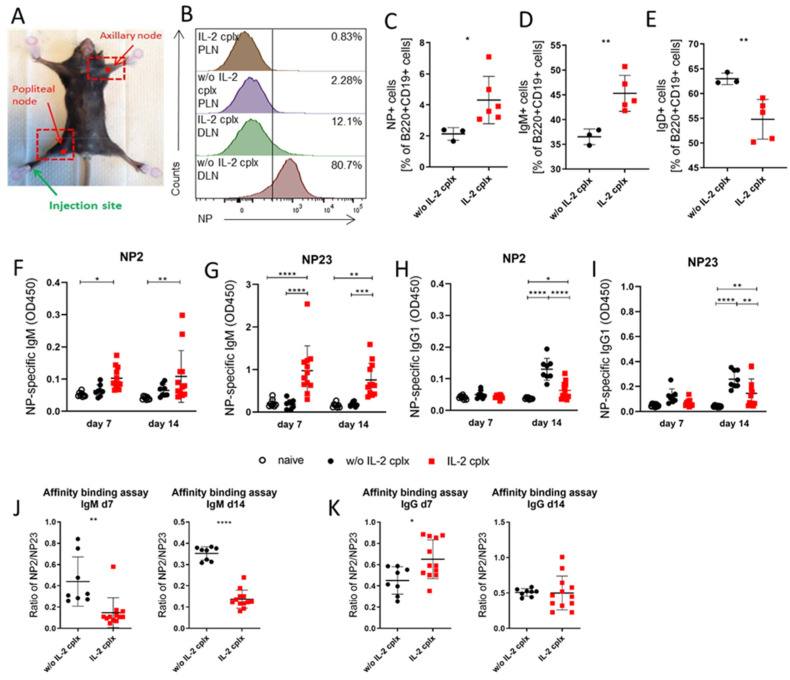
Treatment with JES6-1A12 IL-2 cplx significantly decreases the binding strength of NP-specific IgM Abs and concurrently amplifies that of NP-specific IgG Abs. C57BL/6 mice were i.p. injected with either PBS or JES6-1A12 IL-2 cplx (IL-2 cplx) and immunized i.p. with 10 µg of antigen NP-KLH diluted in 100 µL of Imject alum adjuvant. (**A**–**E**) Spleens (w/o IL-2 cplx *n* = 3, with IL-2 cplx *n* = 6), draining lymph nodes (DLNs; w/o IL-2 cplx *n* = 4, with IL-2 cplx = 6) and peripheral lymph nodes (PLNs; w/o IL-2 cplx *n* = 2, with IL-2 cplx *n* = 2) were analyzed by flow cytometry 14 days post-treatment. (**A**) Injection site (right foot pad), draining lymph node (right popliteal node; DNL) and peripheral lymph node (left axillary node; PLN). (**B**) Representative offset histograms of NP+ cells, gated from B220 + CD19+ cells, in DNL and PLN. (**C**) Proportion of B220 + CD19+ cells that are NP+ in spleen. (**D**,**E**) Percentage of splenic B220 + CD19 + NP+ cells that are IgM+ and IgD+, respectively. (**F**–**K**) Sera were collected on days 7 and 14 post-treatment from non-immunized (*n* = 8) and NP-immunized (w/o IL-2 cplx *n* = 8 and with IL-2 cplx *n* = 12) mice, and NP-specific Abs were determined by ELISA using two different coupling ratios of NP haptens to calculate affinity ability. (**F**,**G**) Bar plots depicting the relative binding abilities of NP-specific IgM Abs to NP2 and NP23 conjugates, respectively. (**H**,**I**) Bar plots depicting the relative binding abilities of NP-specific IgG Abs to NP2 and NP23 conjugates, respectively. (**J**) Ratio of NP2- to NP23-bound serum IgM on days 7 (**left**) and 14 (**right**) post-immunization as a determinant of relative affinity of anti-NP IgM in NP-immunized mice treated with either PBS or IL-2 cplx. (**K**) Ratio of NP2- to NP23-bound serum IgG on days 7 (**left**) and 14 (**right**) post-immunization as a determinant of relative affinity of anti-NP IgG in NP-immunized mice treated with either PBS or IL-2 cplx. In all scatter plots in this figure, each point represents an individual mouse, and bars show means ± SDs. (**B**–**E**) Data representative for 2 independent experiments and (**F**–**K**) data pooled from 2 independent experiments. * *p* < 0.05, ** *p* < 0.01, *** *p* < 0.001 and **** *p* < 0.0001.

To measure the affinity maturation of Abs, we immunized ungrafted control (PBS-injected) and IL-2 cplx-treated mice with the hapten NP conjugated to KLH in alum. The antigen was injected in the right footpad, to induce, via lymphatic drainage, an immune response in the draining ipsilateral popliteal LN (DLN), leaving the contralateral axillary LN as a peripheral LN control (PLN) (Figure 4A). When examined 14 days after antigen injection, the proportion of NP+ B cells in the control group was about 2% in the PLN, up to 80% in the DLN (Figure 4B) and 2% in the spleen (Figure 4C). Interestingly, IL-2 cplx treatment increased the proportion of NP+ B cells in the spleen (4%; Figure 4C) but substantially reduced the proportion in both the DLN (12%) and the PLN (1%), relative to the untreated group (Figure 4B). For the spleen, the increase in the proportion of NP-specific B cells (Figure 4C) was accompanied by an increase in the % of IgM B cells (Figure 4D) but a decrease in IgD+ B cells (Figure 4E).

The titers of NP-specific Abs in the serum of immunized mice were quantitated by binding to BSA protein coupled with NP at a low or high density, NP2 and NP23, respectively. Compared with untreated mice, the levels of NP-specific IgM Abs were significantly increased in IL-2 cplx-treated mice (Figure 4F,G), whereas the levels of IgG were substantially reduced (Figure 4H,I); this finding applied to both NP2 and NP23 binding and was apparent on both day 7 and day 14 after immunization. Significantly, for the IL-2 cplx-treated group, the binding ability of NP-specific IgM Abs on days 7 and 14 post-immunization was reduced 4-fold relative to control immunized mice, indicating a preponderance of low-affinity Abs (Figure 4J). The results for IgG Abs were quite different. Here, treatment with IL-2 cplx led to moderately stronger NP binding on day 7 post-immunization and equivalent binding on day 14 (Figure 4K).

In summary, IL-2 cplx treatment during the immune response to NP primarily induced IgM production with low NP-binding affinity, suggesting an extrafollicular response with limited B-cell maturation. For IgG responses, by contrast, Ab production was low but maintained high-affinity NP binding, indicating a restricted GC response without impaired SHM.

## 4. Discussion

In this study we report impaired GC development in mice that received a fully MHC-mismatched skin graft under IL-2 cplx treatment. As ABMR is still a major issue in clinical solid organ transplantation [35], we think that selective IL-2 therapy that promotes Treg proliferation (amongst other possible immunomodulatory aspects) could not only reduce immunosuppression, as shown previously [12,15], but also prevent long-term graft loss due to ABMR [12]. Our novel insights into the effects of IL-2 cplx treatment on the humoral (allo) immune response represent a critical step toward this goal.

In IL-2 cplx-treated mice, the numbers of GC-phenotype Tfh, Tfr and B cells were all considerably reduced, which correlated with significantly lower numbers of donor-specific IgG, but not IgM. Hence, IL-2 cplx treatment appeared to impair the entry of antigen-stimulated T and B cells into the GC but had minimal qualitative impact on GC responses. Parallel studies with a hapten–carrier affinity maturation assay indicated that the reduced numbers of IgG B cells showed no reduction in binding affinity for the antigen, whereas IgM B cells failed to undergo affinity maturation.

For GC T cells, the development starts when conventional naïve CD4 precursor T cells encounter antigens on dendritic cells in the T-cell zones of lymphoid tissues [36,37]. This extrafollicular interaction drives the responding T cells to synthesize IL-21 and elicit the STAT-3-mediated expression of Bcl-6 along with the upregulation of CXCR5, thus guiding the cells to enter the nascent GC as Tfh cells with a CD4+ CXCR5+PD1+FoxP3-CD25- phenotype. These Tfh cells then drive specific B cells to undergo SHM and produce high-affinity Abs. In parallel with Tfh generation, the antigen-specific TCR stimulation of CD25+ Tregs causes these precursor cells (CD4+CXCR5+PD1+FoxP3+CD25+; pre-Tfr) to downregulate CD25, upregulate Bcl-6 and enter GC as a population of CD4+CXCR5+PD1+ FoxP3+CD25-Bcl-6+ Tfr cells. Although GC Tfh cells are usually defined in the literature as CXCR5+PD-1+ CD4 T cells, emerging evidence suggests that this population is heterogeneous. Therefore, we want to note that the population we are referring to throughout this manuscript may also include non-GC Tfh cells.

The reduction in GC-phenotype T cells in the IL-2 cplx-treated grafted (but not unsensitized) mice appears to be antigen-driven and likely reflects the skewing of primed allospecific T cells away from GC formation towards an extrafollicular fate. The finding that IL-2 cplx treatment failed to affect IgM allo-Ab production is consistent with this opinion.

Since the reduction in GC T-cell numbers by IL-2 cplx applied to both Foxp3+ and Foxp3- cells, the diversion of antigen-specific cells away from GC development involved both T helper and Treg precursor cells. The latter and their pre-Tfr progeny are known to be CD25+ and hypersensitive to IL-2 via high-affinity IL-2Rabg expression [38]. Hence, contact with IL-2 presumably initially expanded these pre-Tfr cells and then, after Bcl-6 induction, drove the cells to differentiate into typical CD25- Tfr cells for GC entry. This scenario may also apply to T helper cells. Thus, contact with antigen plus IL-2 and other cytokines may stimulate some naïve precursor CD4 cells to upregulate CD25, as well as GC markers, and then later foster their differentiation into CD25- Tfh cells.

However, IL-2 clearly also has negative effects on T-cell responses. As mentioned above, IL-2 treatment of grafted mice reduced rather than enhanced the generation of Tfr and Tfh cells, which is in line with the decreased formation of these two subsets seen in mice injected with soluble IL-2 during influenza infection [39] and the direct depletion of autoreactive Tfh cells via low-dose IL-2 in an autoimmune model [40]. By the same token, Tfh and Tfr differentiation is elevated in viral infections after IL-2 blockade [41]. These findings can be attributed to IL-2 signaling, which promotes diversion into extrafollicular T-cell differentiation. Additionally, IL-2 directly inhibits the progression of these cells by blocking their transition from CD25+ precursors. For instance, in mice responding to ovalbumin, previous studies showed that IL-2 cplx injection reduced the percentage of Tfr-lineage cells in the spleen, and notably, this reduction was more marked for CD25- cells than for CD25+ cells [26]. This observation is in agreement with the current finding that IL-2 cplx treatment of allografted mice led to a marked reduction in Tfr-phen cells but with increased CD25 expression on the residual cells. It is also in line with the reciprocal finding that IL-2 blockade during viral infection led to the increase in the Tfr/Tfh ratio [42], thus reflecting enhanced transition of CD25+ Tfr-phen cells into CD25- Tfr cells in the absence of IL-2. Why Tfr cells downregulate CD25 expression in GC is unclear but may reflect a need to prevent the IL-2-mediated upregulation of Blimp-1 [26].

The direct inhibitory effects of IL-2 could also be explained by Treg expansion resulting in the depletion of IL-2 via absorption, thus facilitating Tfr- and Tfh-cell generation. IL-21 synthesis by Tfh stimulates Th precursors to synthesize Bcl-6 rather than Blimp-1 for GC entry [43]. In the studies reported here the inhibitory effect of IL-2 may reflect direct suppression by Tregs and Tfr-lineage cells through cell–cell contact. Thus, despite IL-2 consumption, IL-2-expanded Tregs can directly suppress T-cell function by stripping CD80 and CD86 [44] and peptide–MHC II molecules [45] from APC surfaces. Additionally, Tregs and their progeny release soluble suppressive mediators such as IL-10 [46]. Importantly, in a model of murine systemic lupus erythematosus, it was recently shown that low-dose IL-2 directly depletes autoreactive Tfh cells and prevents auto-Ab formation [40].

We observed that IL-2cplx administration in naïve mice had an opposite effect on T follicular helper (Tfh)-cell and T follicular regulatory (Tfr)-cell accumulation compared with the skin-allograft model. In naïve mice, these T-cell populations expanded, whereas in the allograft model, their expansion was more controlled. This discrepancy may be attributed to the different immune environments and the presence of alloantigens in the allograft model, which could influence the dynamics of Tfh- and Tfr-cell responses. The allograft setting likely induces a more complex immune response, where regulatory mechanisms are more actively engaged to maintain tolerance and prevent rejection. Further investigation is needed to elucidate the specific factors and signaling pathways that contribute to these opposing phenotypes.

Notably, the effects of IL-2 cplx treatment on IgM and IgG Ab production differed significantly both in skin-grafted mice and those subjected to hapten–carrier immunization. IL-2 cplx treatment did not impair specific IgM Ab production; in fact, IgM levels increased in the hapten–carrier system. However, the binding affinity of IgM anti-NP Abs was considerably reduced, indicating minimal SHM. High-affinity IgM Ab generation requires GC differentiation and SHM [34], similar to IgG. Therefore, the data suggest that IL-2 cplx treatment promotes the production of low-affinity IgM Ab via extrafollicular pathways. Regarding the discrepancy between NP+ B cells in the spleen but not the DLN, we hypothesize that this may be due to the activation of MZ B cells or B1 B cells, which are abundant in the spleen but less prevalent in the DLN. The spleen’s unique microenvironment and its role in filtering blood-borne antigens could contribute to the preferential activation and expansion of these B-cell subsets at this site.

In contrast to IgM, IgG Ab production was strongly inhibited by IL-2 cplx treatment in both models. In the skin-graft model, this inhibition correlated with a scarcity of GC B cells, consistent with our previous findings [12]. Surprisingly, however, IL-2 cplx treatment increased the binding strength of IgG Ab during early (day 7) responses in the hapten–carrier model. This result may be explained by the role of Tfr cells in the GC reaction. Tfr cells are thought to selectively regulate B-cell responses by targeting low-affinity and self-reactive B cells for elimination [47,48,49]. Indeed, there is growing evidence that Tfr cells enhance the quality of IgG responses to foreign antigens by promoting affinity maturation. Here, neuritin plays a key role: the selective depletion of its synthesis by Tfr cells leads to the accumulation of self-reactive B cells and impaired IgG responses to foreign antigens [50]. Thus, there is the possibility that Tfr and also Treg are acting directly on B cells in our models, a mechanism previously shown in other systems [51,52,53,54]. However, the role of B-cell subsets warrants additional studies, as we cannot outright preclude any possible impact of IL-2 cplx on B cells.

Although B220+Fas+GL7+Bcl6+ is used to define GC B cells via flow cytometry by us and others, this phenotype alone does not always indicate that these B cells are in an actual germinal center; further experiments showing actual GC morphology could provide additional insights to supplement the findings from this study.

The mechanisms of IL-2 cplx treatment shown in this study are based on previously published protocols aiming at tolerance induction in an experimental setting [10,12]. Pre-treatment might not be possible in the clinical setting (with the exception of living donation), and further studies elucidating the efficacy of IL-2 cplx treatment in peri- or post-transplant protocols might be warranted. However, a recent publication used a similar approach of IL-2-induced Treg expansion, starting treatment on the day of transplantation with a similar outcome with regard to the prolongation of allograft survival and reduction in DSA development [15].

Given that the effects of IL-2cplx in the skin-allograft model are transient without additional immunosuppressive or anti-inflammatory treatment, with graft rejection starting within 9–10 days after the last dose, it is possible that the ability of IL-2cplx to suppress DSA response may wane over time. This could result in the detection of anti-donor antibodies in the recipient mouse as the effect diminishes. While our current models do not directly address this question, we speculate that the sustained or repeated administration of IL-2cplx might be necessary to maintain the long-term suppression of the DSA response. Notably. in our previous work, prolonged IL-2cplx treatment combined with rapamycin and short-term anti IL-6 was able to inhibit the effect. Although IL-2cplx treatment results in the production of high-affinity antibodies only in lower quantities, these antibodies could still pose a risk for graft rejection in transplant patients. Therefore, combination therapies targeting both T- and B-cell responses, such as B-cell depletion or co-stimulation blockade, should be considered to enhance the efficacy of IL-2cplx treatment and achieve long-term graft survival and immune tolerance. Future studies are needed to explore the optimal dosing regimen and frequency to achieve prolonged immune tolerance and prevent antibody-mediated rejection.

## 5. Conclusions

In summary, the potential of IL-2 cplx to diminish allospecific humoral responses and prevent sensitization might offer a new perspective in transplantation medicine. While the preclinical results are promising, successful translation will require further validation. This therapeutic approach holds promise for improving the long-term survival of transplant recipients by addressing a critical unmet need: the effective treatment of ABMR. Given that there are currently no FDA-approved treatments specifically for ABMR, IL-2 cplx-based immunomodulation shows great potency for not only the induction of cellular immunosuppression but also the prevention of the humoral alloresponse. Overall, IL-2 complex treatment shows significant promise in selectively expanding Tregs, promoting immune tolerance and potentially reducing the burden for lifelong immunosuppression. However, translating these findings into NHP models and ultimately clinical practice will require overcoming additional challenges, including heterologous immunity and the pre-sensitization status of patients.

## Figures and Tables

**Table 1:** Markers for the phenotypic characterization of B- and T-cell subpopulations.

	Phenotypes	References
**B-cell subpopulations**		
Mature B cells	B220 + IgM + IgD + CD23+	[17]
GC B cells	B220 + GL7 + Fas + Bcl-6+	[18,19]
Marginal zone B cells	B220 + IgM + CD1d + CD23−	[20]
Plasma cells	B220-CD19-CD3-CD138 + Blimp-1+	[21,22]
**T-cell subpopulations**		
Tregs	CD4 + CD25 + FoxP3+	[23]
GC T cells	CD4 + CXCR5 + PD-1 + Bcl-6+	[24]
Tfh	CD4 + CXCR5 + PD-1 + Bcl-6 + FoxP3−	[25]
Tfr	CD4 + CXCR5 + PD-1 + FoxP3 + CD25−	[26]
Tfr-phenotype (Tfr-phen)	CD4 + CXCR5 + PD-1 + FoxP3+	[27]
Pre-Tfr	CD4 + CXCR5 + PD-1 + FoxP3 + CD25+	[26]

## Data Availability

Correspondence, raw data and material requests should be addressed to N.P. (nina.pilat@meduniwien.ac.at).

## Supplementary Materials

The following supporting information can be downloaded at: https://www.mdpi.com/article/10.3390/cells14141086/s1, Figure S1: Influence of JES6-1A12 IL-2 cplx on T cell subsets in the murine spleen; Figure S2: Influence of JES6-1A12 IL-2 cplx on B cell subsets in the murine spleen; Figure S3: Gating strategy for T and B cell subpopulations and DSA IgG subpopulations.

## Abbreviations

The following abbreviations are used in this manuscript:

ABMR	antibody-mediated rejection
DLN	draining lymph node
DSA	donor-specific antibody
EAE	experimental autoimmune encephalomyelitis
GC	germinal center
GVHD	graft-versus-host disease
KLH	keyhole limpet hemocyanin
MST	median survival time
MZ B cells	marginal zone B cells
NP	4-Hydroxy-3-nitrophenylacetyl
PLN	peripheral lymph node
SHM	somatic hypermutation
Tfh	T follicular helper cells
Tfr	T follicular regulatory cells
Tfr-phen	T follicular regulatory phenotype
Tregs	regulatory T cells
