# Supplementary material for: IL-2 Complex Therapy Mitigates Humoral Rejection of Fully Mismatched Skin Allografts by Inhibiting IgG Alloantibody Formation
Source: Cells. 2025 Jul 16;14(14):1086. doi: 10.3390/cells14141086 (PMC12294099; doi:10.3390/cells14141086)
Supplement: Supplementary file 1 [file cells-14-01086-s001.zip › cells-3721984-supplementary.pdf]

SUPPLEMENTARY FIGURES & LEGENDS

Supplementary Figure S1

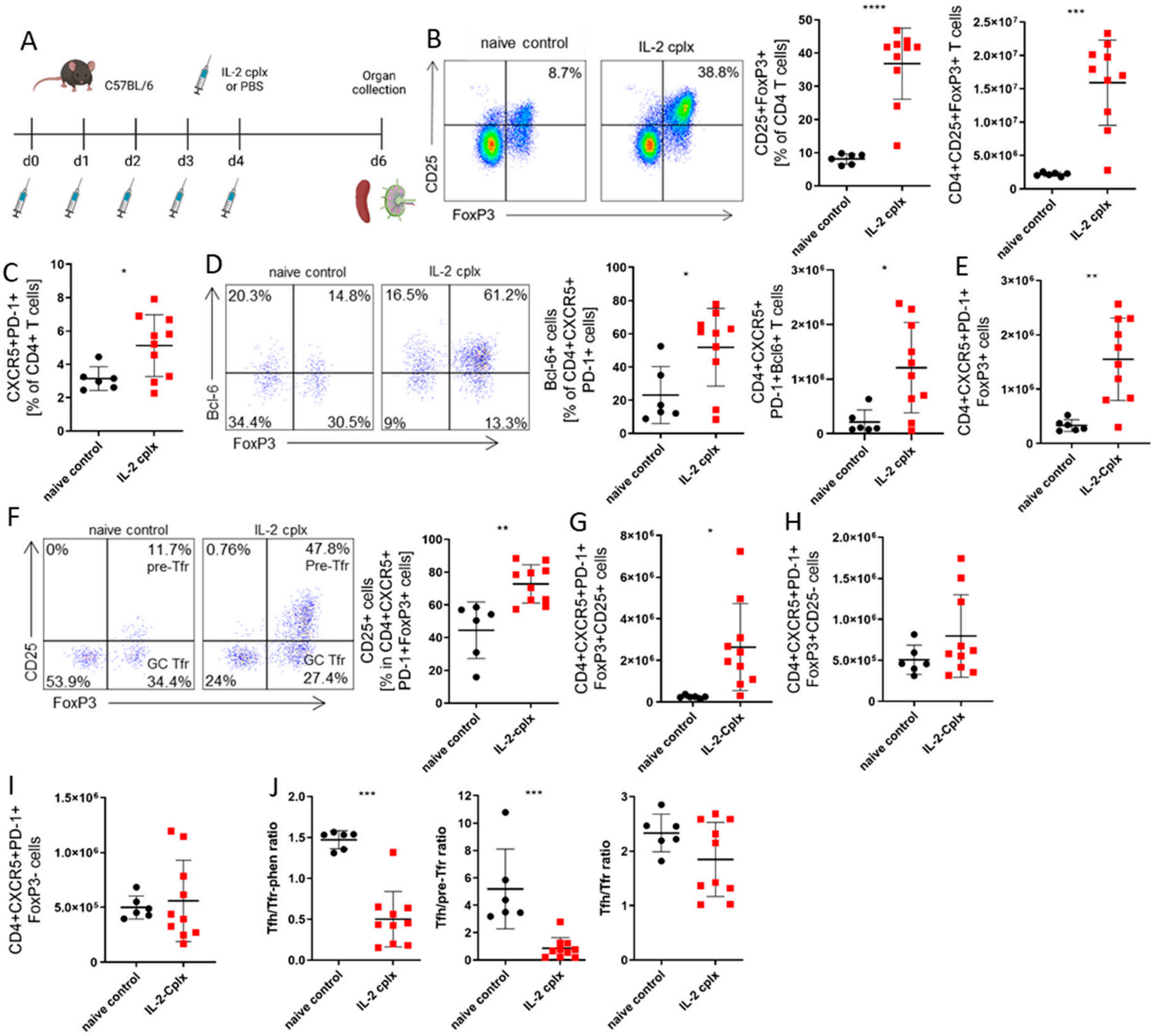

Supplementary Figure Legend S1: Influence of JES6-1A12 IL-2 cplx on T cell subsets in the murine spleen

C57BL/6 mice received i.p. injections of either PBS (naïve control, n=6) or JES6-1A12 IL-2 cplx (IL-2 cplx, n=10) for five consecutive days and were sacrificed on day 6. (A) Schematic illustration of the experimental setup. (B) Representative dot plots of CD25 and FoxP3 staining, gated on CD4+ T cells, from splenocytes of control (left) and IL-2 cplx-treated (right) mice. Scatter plots depict the frequency and absolute counts of Treg cells (CD4+CD25+FoxP3+) from control and IL-2 cplx-treated splenic lymphocytes. (C) Scatter plots show the percentage of CXCR5+PD1+ cells within CD4 T cells of control and IL-2 cplx-treated mice. (D) Representative dot plots show intracellular Bcl-6 and intracellular FoxP3 staining, gated on CD4+PD1+CXCR5+ cells while scatter plots show Bcl-6 expression in CD4+CXCR5+PD-1+ T cells and absolute counts of GC T cells (CD4+CXCR5+PD1+Bcl-6+) for control and IL-2 cplx-treated spleens. (E) Absolute count of FoxP3 expressing GC T cells of control and IL-2 cplx-treated splenic lymphocytes (Tfr phen). (F) Representative dot plots show expression of CD25 and FoxP3, gated on CD4+CXCR5+PD1+ cells, and scatter plots showing the proportion of CXCR5+PD1+FoxP3+ cells that express CD25+ in control and IL-2 cplx-treated spleens. (G) The absolute count of pre-Tfr cells (CD4+CXCR5+PD1+FoxP3+CD25+) and (H) Tfr cells

(CD4+CXCR5+PD1+FoxP3+CD25-), whereas (I) shows the absolute number of Tfh cells (CD4+CXCR5+PD1+FoxP3-). (J) The ratio of splenic Tfh to Tfr-phen, pre-Tfr or Tfr cells is shown. In all scatter plots in this figure, each point represents an individual mouse and bars show mean±SD.

Supplementary Figure S2

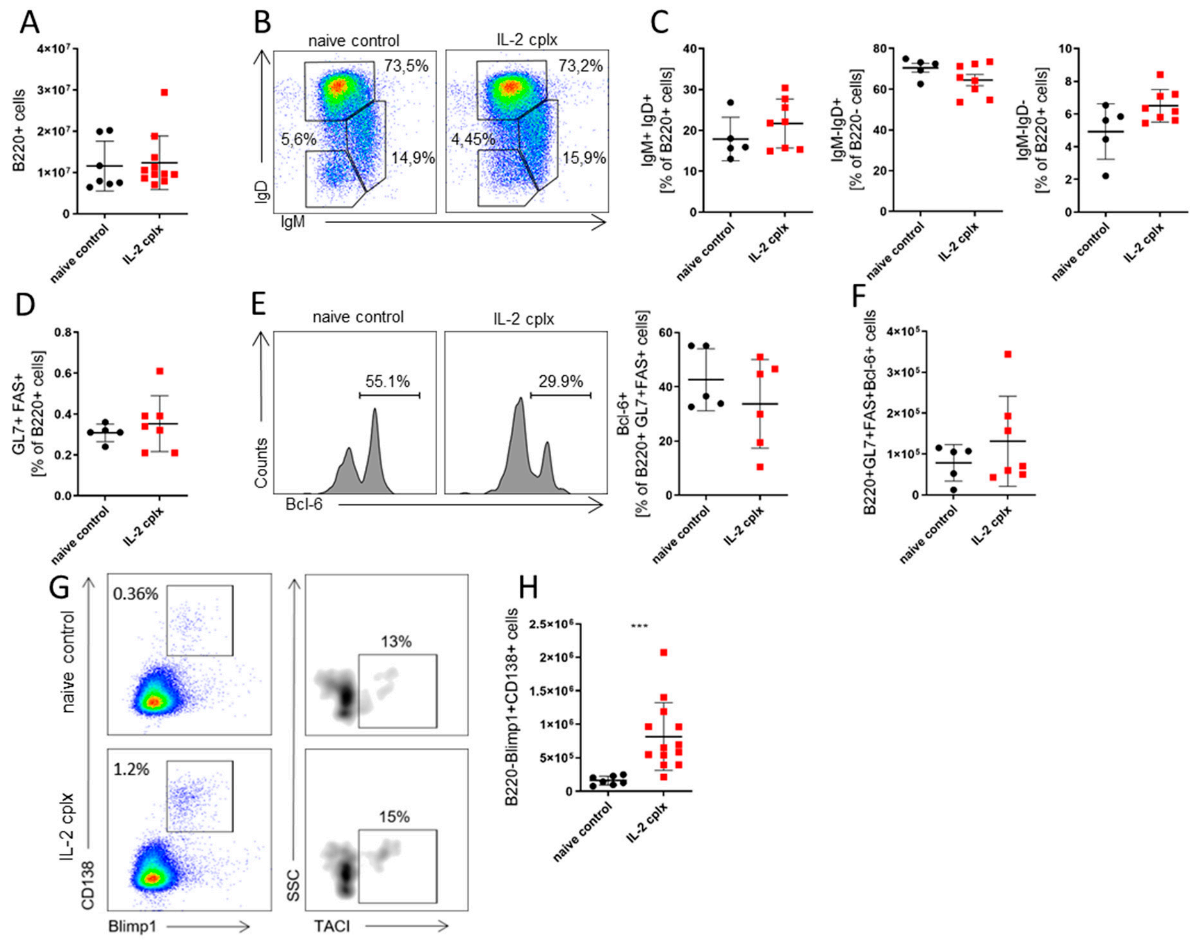

Supplementary Figure Legend S2: Influence of JES6-1A12 IL-2 cplx on B cell subsets in the murine spleen
C57BL/6 mice received intraperitoneal injections of either PBS (naïve control, n=7) or JES6-1A12 IL-2 cplx (IL-2 cplx, n=13) for five consecutive days and were sacrificed on day 6. (A) Scatter plots show the absolute cell count of B220+ cells, gated on splenic lymphocytes, from control and IL-2 cplx-treated mice. (B) Representative dot plots show anti-IgM against anti-IgD staining, gated on B220+ splenocytes, recovered from control and IL-2 cplx-treated mice. (C) Scatter plots show the proportion of IgM+IgD+, IgM-IgD+ or IgM-IgD- B cells, respectively. (D) shows the proportion of B220+ splenocytes that are GL7+Fas+. (E) The proportion of B220+GL7+Fas+ cells that express intracellular Bcl-6 is shown in representative histograms (left) and a scatter plot (right) from naive control and IL-2 cplx-treated mice. (F) Scatter plots show the absolute count of B220+GL7+Fas+Bcl-6+ cells in the spleens of control and IL-2-treated mice. (G) Representative FACS plots show CD138 against intracellular Blimp1, gated on B220-CD19-CD3- splenocytes (left) and TACI staining of B220-CD19-CD3-CD138+Blimp1+ cells (right) from control (top row) and IL-2 cplx-treated (bottom row) adult mice. (H) shows the absolute cell count of B220-CD19-Blimp1+CD138+ cells. In all scatter plots in this figure, each point represents an individual mouse and bars show mean±SD.

Supplementary Figure S3

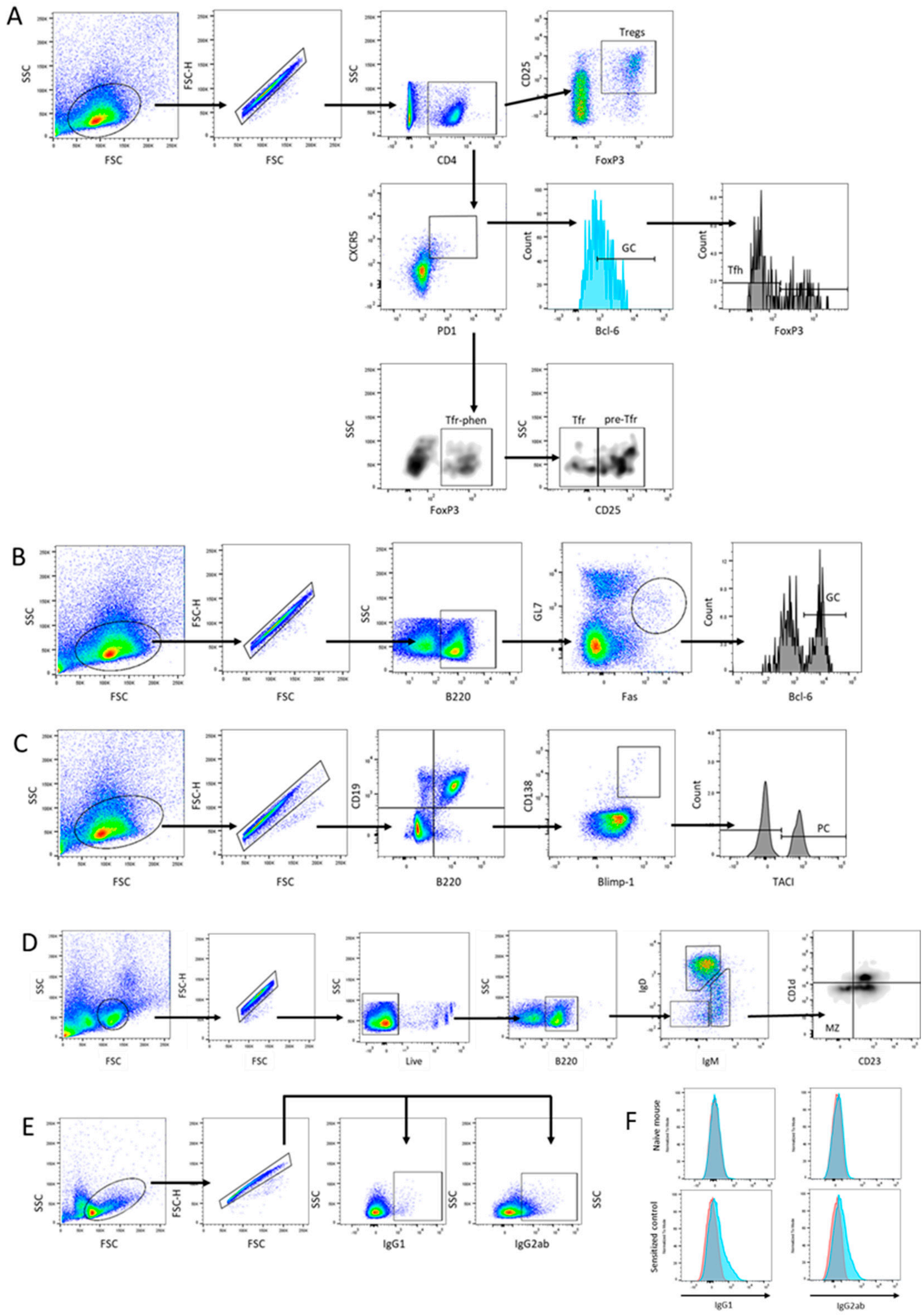

Supplementary Figure Legend S3: Gating strategy for T and B cell subpopulations and DSA IgG subpopulations

Logic and manual gating strategy for characterization T and B cell subpopulations. Cells (single cell suspensions freshly prepared from spleen, lymph nodes or thymus) were plotted forward scatter area vs side scatter area to determine the lymphocyte population (plots on the left) and subsequently gated to include singlets and markers of interest to characterize (A) Tregs, Tfh, Tfr-

phen, pre-Tfr and Tfr T cell populations, (B) GC B cells, (C) PCs, (D) MZ and mature B cells and (E) donor-specific IgG subtypes IgG1 and IgG2ab. (F) Controls for IgG expression are shown (red indicates serum incubation with recipient type thymocytes (negative control, B6 mice), blue indicates serum incubation with donor type thymocytes (Balb/c mice) = DSA positive cells).
